# Could cannabinoids provide a new hope for ovarian cancer patients?

**DOI:** 10.1002/prp2.1122

**Published:** 2023-08-01

**Authors:** Rimanee Kaur, Farideh A. Javid

**Affiliations:** ^1^ Department of Pharmacy, School of Applied Sciences University of Huddersfield Huddersfield UK

**Keywords:** cannabinoids, non‐psychoactive cannabinoids, ovarian cancer

## Abstract

It is known that gynecological cancers remain a worldwide problem and as shown by the statistics, there is a need for new gynecological cancer treatments. Cannabinoids, the pharmacologically active compounds of the *Cannabis sativa* plant, have been used for many centuries by individuals as a symptomatic treatment to alleviate pain, nausea, vomiting, and to help stimulate appetite. Research has revealed that cannabinoids also exert anti‐cancer activity such as anti‐proliferative and pro‐apoptotic effects through a variety of mechanisms. There is significant value in the development of these compounds as anti‐cancer therapies in clinical practice as they do not produce the typical toxic side effects that exist with conventional therapies and recent clinical trials have shown their great tolerability by patients at high doses. Cannabinoids can induce psychoactive effects that could limit their progression. Therefore, non‐psychoactive cannabinoids are attracting pharmacological interest due to their inability to produce psychological effects. Recent studies have focussed on non‐psychoactive cannabinoids in ovarian cancer and have revealed promising pre‐clinical results that indicate that these compounds may have potential benefits in the treatment of these cancers. However, there are still unanswered questions and research gaps that need to be addressed. This review summarizes the current understanding of this topic and identifies the current gaps in knowledge that provide a useful direction for future work.

## Graphical abstract

A summary of the altered signalling pathways and proteins leading to evasion of cell death via apoptosis, cell cycle arrest and autophagy in ovarian cancer that are affected by cannabinoids.

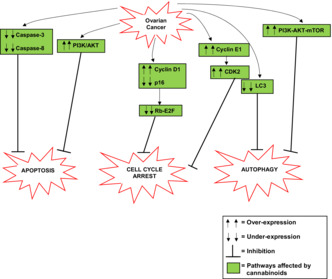

**Abbreviations**
2‐AG: 2‐arachidonoylglycerol
2‐AGE: 2‐arachidonoyl glyceryl ether
AEA‐ANANDAMIDE: N‐arachidonoylethanolamine
AKT: Protein Kinase B
CB_1_
: Cannabinoid receptor 1
CB_2_
: Cannabinoid receptor 2
CBC: Cannabichromene
CBCV: Cannabichromevarin
CBD: Cannabidiol
CBDV: Cannabidivarin
CBG: Cannabigerol
CBGM: Cannabigerol monoethyl ether
CBGV: Cannabigerovarin
CBL: Cannabicyclol
CBV: Cannabivarin
CDK2: Cyclin‐dependent kinase 2
CNS: Central Nervous System
ECS: Endocannabinoid System
EGF: Epithelial Growth Factor
EGFR: Epithelial Growth Factor Receptor
ER: Endoplasmic Reticulum
ERK: Extracellular Signal‐Regulated Kinase
FAAH: Fatty acid amide hydrolase
FSH: Follicle‐stimulating Hormone
GnRH: Gonadotropin‐releasing hormone
GPR55: G protein‐coupled receptor 55
HDAC: Histone deacetylase
IAP: Inhibitors of Apoptosis Proteins
LH: Luteinizing hormone
MAGL: Monoacylglycerol lipase
mTOR: Mammalian target of rapamycin
NADA: N‐arachidonoyldopamine
NAGly: N‐arachidonylglycine
OA: Oleic acid amide
PI3K: Phosphatidylinositol 3‐kinase
PPARs: Peroxisome proliferator‐activated Receptors
ROS: Reactive oxygen species
THC: Δ^9^‐tetrahydrocannabinol
THCV: Tetrahydrocannabivarin
TRP: Transient receptor potential
TRPV1: Transient receptor potential vanilloid subtype 1
XIAP: X‐linked inhibitor of apoptosis protein

## 1 INTRODUCTION

Gynecological cancers are cancers of the female reproductive system and consist of cancers of the cervix, ovaries, vagina, vulva, and uterus.^1^ Among gynecological cancers, ovarian cancer has the highest morbidity and mortality rates.^2^ Globally, the number of patients diagnosed in 2018 with gynecological cancers was >295 000 for ovarian. In 2018, approximately 185 000 women died from ovarian cancer.^3^ It is clear that there are unfavorable outcomes and that today, there are still unmet clinical needs.

Despite improvements in treatment strategies (Table 1), tumor recurrence, drug resistance, and toxic effects remain a major challenge, indicating the need for a new approach to the treatment of ovarian cancer. Recently, cannabinoids have gained attention as potential anti‐cancer therapies due to their ability to modulate the intracellular signaling pathways involved in cancer progression.^4^ However, the presence of psychoactive effects of cannabinoids could limit their progress in this field, therefore recent studies have highlighted the value of non‐psychoactive cannabinoids such as cannabidiol (CBD).^5^ CBD has been shown to demonstrate a broad array of anti‐carcinogenic properties, such as anti‐proliferative action toward breast cancer cells,^6^ anti‐invasive and anti‐metastatic effects on lung cancer cells^7^ as well as induction of apoptosis.^8^

**TABLE 1:** Current treatment strategies for ovarian cancer.^a^

	Surgery	First‐line chemotherapy	Maintenance treatment
Stage I	Yes	Only for stage Ic or grade 3—six cycles of carboplatin	Not recommended
Stage II	Yes	Paclitaxel plus platinum‐based compound or platinum‐based therapy alone (cisplatin or carboplatin)	Not recommended
Stage III	Yes	Paclitaxel plus platinum‐based compound or platinum‐based therapy alone (cisplatin or carboplatin)	Olaparib plus bevacizumab or niraparib alone Olaparib for BRCA mutation‐positive ovarian cancer
Stage IV	Yes	Paclitaxel plus platinum‐based compound or platinum‐based therapy alone (cisplatin or carboplatin)	Olaparib plus bevacizumab or niraparib alone Olaparib for BRCA mutation‐positive ovarian cancer

^a^ National Institute for Health and Care Excellence (NICE) Guidance Ovarian Cancer: Recognition and Initial Management (2011).^9^

In this review, the current knowledge on ovarian cancer treatment and the rationale for new therapies are discussed. In addition, this review provides a summary of the endocannabinoid system, the action of cannabinoids in cancer settings, and their anti‐tumor properties, as well as the effects of non‐psychoactive cannabinoids in ovarian cancer. Finally, recommendations for future work will be included in relation to their potential as a novel gynecological cancer treatment.

## 2 CURRENT CLINICAL STATUS OF OVARIAN CANCER AND RATIONALE FOR NOVEL THERAPIES

The current standard of care for patients with ovarian cancer is a combination of optimal cytoreductive surgery and platinum‐based chemotherapy.^10^ Ovarian cancer usually metastasizes within the peritoneal cavity within the abdomen first and in these cases, surgical debulking is used to inform staging and adjuvant therapy. Key advances in radical surgery and chemotherapy strategies have led to improved, yet modest, clinical outcomes. Despite this, there remains a significant risk of resistance to drug therapy and tumor recurrence. Due to the site of presentation and the shortage of promising screening tools for early‐stage detection, ovarian cancer often presents at a late stage, resulting in a poor 5‐year survival rate for these patients even with optimal care.^10^ Patients with stage III or IV disease have a 70%–75% chance of recurrence within 2 years of diagnosis. Treatment options are less effective at each recurrence, highlighting the need for novel maintenance therapies (Table 1).^10^ Further treatments after relapse are more intense, resulting in increased drug toxicity, drug resistance, and financial burden to patients with poor quality of life.^11^ Thus, there is a clear need for new and improved therapies to address the issues that remain with current conventional treatments.

## 3 THE HISTORY OF CANNABINOIDS

The first discovered and most important source of cannabinoids was the *Cannabis sativa* plant, which has been used as a herbal remedy for centuries. The earliest archeological evidence of the use of medicinal cannabis use was in ancient China where it was recommended for rheumatic pain, constipation, disorders of the female reproductive tract, and malaria, among other conditions.^12^ In Western medicine, the use of cannabis was introduced by William B. O'Shaughnessy (an Irish physician) and Jacques‐Joseph Moreau (a French psychiatrist) in the mid‐19th century. They described positive effects of cannabis preparations on pain, vomiting, convulsions, rheumatism, tetanus, and mental ability. From 1851, cannabis was recognized as a medicine in the United States (US) Pharmacopeia, in the form of tinctures, extracts, and resins. However, at the beginning of the 20th century, cannabis use decreased due to its increased use as a recreational drug, abuse potential, variability in the quality of herbal material, unidentified active compounds, and alternative medications with known efficacy being introduced to treat the same symptoms.^13^, ^14^ In 1941, as the result of many legal restrictions, cannabis was considered to be in the same group as other illicit drugs and was removed from the American Pharmacopeia.^14^ Consequently, the exploration of medicinal uses of cannabis considerably slowed down for more than half a century.

However, in the late 20th century, studies conducted on cannabis led to the identification and extraction of pharmacologically active components named cannabinoids.^15^ The principal psychoactive constituent (or cannabinoid) of the cannabis plant is Δ^9^‐tetrahydrocannabinol (THC). This compound was first isolated, identified, and synthesized in 1964.^16^ Its discovery subsequently led to the identification of cannabinoid receptors and their endogenous ligands. Various therapeutic actions of these compounds have been reported and were thought to be mediated through the endocannabinoid system (ECS).^17^ Current legislative changes that allow cannabis for medical and/or recreational use, the progress in scientific research, and public awareness of the benefits of medicinal cannabis have all contributed to the increasing interest in the therapeutic potential of cannabinoids.^18^, ^19^

## 4 TYPES OF CANNABINOIDS

As of 2020, over 150 cannabinoids have been identified.^20^ Cannabinoids comprise of (a) the active compounds of the *Cannabis sativa* plant (known as phytocannabinoids), (b) endogenous cannabinoids that are produced in our body (known as endocannabinoids), and (c) synthetic cannabinoids.^21^

Phytocannabinoids occur naturally in significant quantities in the cannabis plant and are concentrated in a viscous resin that is produced in glandular structures known as trichomes. Of all phytocannabinoids discovered so far, THC and CBD are the most abundant.^22^ Other phytocannabinoids include cannabigerol (CBG), cannabichromene (CBC), cannabicyclol (CBL), cannabivarin (CBV), tetrahydrocannabivarin (THCV), cannabidivarin (CBDV), cannabichromevarin (CBCV), cannabigerovarin (CBGV), and cannabigerol monoethyl ether (CBGM).^21^ As well as their analgesic, neuroprotective, and anxiolytic effects, studies have highlighted the therapeutic potential of phytocannabinoids for a variety of widespread skin conditions including acne, psoriasis, atopic dermatitis, and pruritis.^23^

Endocannabinoids are produced in our body and consist of lipid molecules containing long‐chain polyunsaturated fatty acids, amides, esters, and ethers that bind to cannabinoid receptors.^24^ Endocannabinoids act mainly as neuromodulators that affect the release of various neurotransmitters in the peripheral and neural tissues.^25^ They also play an important role in inflammation, insulin sensitivity, and fat and energy metabolism.^26^ Two of the best‐characterized endocannabinoids are N‐arachidonoylethanolamine (AEA‐anandamide) and 2‐arachidonoylglycerol (2‐AG), which are derived from arachidonic acid and affect our mood, appetite, pain sensation, inflammatory response, and memory.^27^, ^28^

Synthetic cannabinoids such as the CB_2_ synthetic agonist, JWH‐133, and the CB_1_ and CB_2_ agonist, WIN‐55 have been widely used as a pharmacological agent, both in vitro and in vivo to obtain a more detailed understanding of cannabinoid action in order to evaluate their potential clinical use.^29^, ^30^

## 5 THE ENDOCANNABINOID SYSTEM (ECS)

Historically, the two main cannabinoid receptors: cannabinoid receptor 1 (CB_1_) and cannabinoid receptor 2 (CB_2_), the main endocannabinoids: AEA‐anandamide and 2‐AG and the enzymes that produce and degrade these endocannabinoids have been known as ECS.^31^

In recent years, further components have widened this original definition of the ECS. These components consist of newly discovered endocannabinoid receptor ligands such as 2‐arachidonoyl glyceryl ether (noladin ether, 2‐AGE), O‐arachidonoylethanolamine (virodhamine), N‐arachidonoyldopamine (NADA) and oleic acid amide (oleamide, OA). In addition, newly discovered receptors such as G protein‐coupled receptor 55 (GPR55) and PPARs.^32^ However, other receptors have been recognized to participate in cannabinoid signaling. For example, it has been discovered that cannabinoids can affect a subset of transient receptor potential (TRP) channels.^33^ TRP subfamilies have been found to contain channels that can be modulated by endogenous, phytogenic and synthetic cannabinoids. TRP channels from these subfamilies have been reported to mediate cannabinoid activity.^33^ In addition to receptors and their cannabinoid ligands, the ECS encompasses several enzymes that regulate the biosynthesis and degradation of endocannabinoids. The catabolic enzyme primarily responsible for the degradation of AEA‐anandamide is fatty acid amide hydrolase (FAAH), while the main enzyme responsible for the degradation of 2‐AG is monoacylglycerol lipase (MAGL).^31^

The ECS plays an important role in the organism's physiology. Dysregulation of the ECS due to variation in the expression and function of cannabinoid receptors, enzymes, or the concentration of endocannabinoids, has been associated with several diseases, such as neurodegenerative disorders, multiple sclerosis, inflammation, epilepsy, schizophrenia, glaucoma, cardiovascular diseases, obesity, and cancer.^34^, ^35^

## 6 CANNABINOID RECEPTORS

Insights into the mechanism of action of phytocannabinoids led to the identification of two G protein‐coupled receptors, CB_1_ and CB_2._ ^36^, ^37^ CB_1_ is mainly expressed in the human CNS and is the main receptor responsible for the psychotropic effects of THC.^38^, ^39^ Although to a lesser extent, CB_1_ receptors are also expressed at peripheral tissue sites where they aid in the regulation of local tissue functions.^39^ CB_1_ expression has been reported in adipose tissue, skeletal muscle, bone, skin, eye, reproductive system, and several types of cancer cells.^40^ A principal role for CB_1_ receptors is to inhibit neurotransmitter release. Strongly associated with GABAergic (inhibitory) and glutamergic (excitatory) cells, activation of CB_1_ receptors inhibits the release of GABA and glutamate, respectively.^41^ This decrease in excitability and neurotransmitter release may underlie some of the psychoactive and anti‐convulsant action of cannabinoids.

In contrast, CB_2_ receptors are predominantly expressed in peripheral tissues, such as the immune system, where they modulate immunological function, cell migration, and cytokine release.^39^, ^42^ However, CB_2_ receptor expression has also been detected in the brain, however to a much lower extent compared to the immune system or the level of CB_1_ expression.^39^ CB_2_ activation is associated with neurodefense functions, ensuring the maintenance of bone mass and reduction of inflammation.^43^

In addition to the first discovered cannabinoid receptors CB_1_ and CB_2_, other cannabinoid receptors have since been identified that have responded to cannabinoid ligands, thus suggesting the existence of additional cannabinoid receptors.^44^ GPR55 has been identified as a novel cannabinoid receptor and has shown to interact with and be modulated by endogenous, plant, and synthetic cannabinoid ligands. AEA‐anandamide, the predominant circulating endocannabinoid, has been shown to activate GPR55 with a potency equivalent to that activating CB_1_ and CB_2_ receptors, demonstrating that this ligand has the potential to influence signaling by all three receptors equally.^45^ In addition, Overton et al.^46^ identified GPR119 as a cannabinoid receptor and it has been shown to be activated by endocannabinoids. 2‐AG has also been identified as a GPR119 agonist.^47^ GPR18 has been identified as a candidate cannabinoid receptor, but its classification is controversial. Several cannabinoid ligands have been described to be active as agonists or antagonists.^48^ N‐arachidonylglycine (NAGly) has been identified as an endogenous ligand for GPR18. However, NAGly does not have activity on the classical cannabinoid receptors CB_1_ and CB_2._ ^49^ Therefore, it is difficult to determine whether GPR18 is a cannabinoid receptor.

## 7 CANNABINOIDS AND THEIR ANTI‐TUMOR EFFECTS

Cannabinoids have an established role in exerting palliative effects in cancer patients and have been used to alleviate nausea, vomiting, pain and to help stimulate appetite.^5^, ^50^ Besides from providing symptomatic treatment for cancer patients, cannabinoids have been shown to exert anti‐tumor actions through modulation of the intracellular signaling pathway implicated in cancer progression.^4^, ^5^ The first report on anti‐proliferative properties of cannabinoids was in 1975 when Munson et al.^51^ demonstrated that THC inhibits lung adenocarcinoma cell growth of in vitro cell lines and in murine models after oral administration. Cannabinoids can exert anti‐tumor effects directly through the inhibition of cell proliferation and induction of apoptosis or indirectly through the inhibition of angiogenesis, invasion, and metastasis.^52^ Numerous studies using synthetic/endo‐/phyto‐cannabinoids and ECS regulators in various cancer cell lines support this notion.^53^ The anti‐tumor effects of cannabinoids have also been observed in various animal tumor models.^52^

Endocannabinoids such as AEA‐anandamide have been found to have anti‐proliferative effects in prostate carcinomas. Through activation of CB_1_, AEA‐anandamide inhibited EGF‐induced proliferation of prostate carcinoma cells by decreasing the expression of the EGF receptor (EGFR) and increasing the production of ceramide (a powerful tumor suppressor).^54^ Phytocannabinoids such as THC have shown to reduce tumor proliferation and lung metastases, inhibit angiogenesis and cause apoptosis in a mouse model of ErbB2‐driven metastatic breast cancer. Caffarel et al. showed that these anti‐tumor actions relied at least partially on the inhibition of the pro‐tumorigenic AKT pathway, the signal transduction pathway that promotes cell growth, cell survival, and proliferation.^55^ This shows that cannabinoids can modulate certain pathways involved in cancer development and exert their anti‐tumor effects at the intracellular signaling level. Synthetic cannabinoids such as WIN‐55 have also shown anti‐proliferative effects on tumor progression.^56^

As cannabinoids are generally well tolerated and do not produce the typical toxic effects of conventional chemotherapy, there is considerable merit in their development as potential anti‐cancer therapies. However, the presence of psychoactive effects of cannabinoids could limit their progress in this field. Unfortunately, THC‐based drugs produce both therapeutic and undesirable psychotropic actions by activating CB_1_ receptors in the CNS. However, other cannabinoids such as CBD are devoid of the typical psychological effects. CBD constitutes up to 40% of cannabis extracts with pharmacological effects without producing undesirable psychoactive side effects.^57^ Non‐psychoactive cannabinoids have gained attention due to preclinically established anti‐cancer properties and a favorable risk–benefit profile. Recent studies have indicated the value of non‐psychoactive cannabinoids such as CBD.^5^

## 8 NON‐PSYCHOACTIVE CANNABINOIDS AND THEIR ANTI‐TUMOR ACTIVITY

CBD has demonstrated a broad array of anti‐carcinogenic properties. In glioma cells, CBD has been shown to successfully induce tumor cell death, inhibit cell migration and invasion in vitro, decrease tumor size, vascularization, growth, and weight, and induce tumor regression in vivo.^8^, ^58^, ^59^, ^60^, ^61^, ^62^, ^63^, ^64^ CBD induced anti‐proliferative effects on breast cancer cells through a variety of mechanisms including apoptosis, autophagy, and cell cycle arrest.^6^, ^65^, ^66^ In aggressive breast cancer in vivo and in vitro, CBD has inhibited migration, invasion, and metastasis.^6^, ^67^, ^68^, ^69^ Moreover, in lung cancer cells, CBD has demonstrated anti‐invasive and anti‐metastatic effects.^7^ In leukemias/lymphomas, CBD has mediated cell death by the mechanism of apoptosis.^70^, ^71^, ^72^, ^73^ In prostate cancer cells, CBD has induced anti‐proliferative effects and apoptosis‐mediated cell death via the intrinsic pathway.^74^, ^75^

CBG, another non‐psychoactive cannabinoid has also been shown to exhibit anti‐tumor properties in carcinomas. A recent study by Lah et al.^76^ was the first study to report the anti‐tumor effects of CBG in glioblastoma. They found that CBG effectively impaired the relevant hallmarks of glioblastoma progression and inhibited the invasion of glioblastoma cells. Borrelli et al. investigated whether CBG protects against colon tumourigenesis. The study showed that CBG stimulated ROS production, promoted apoptosis, and reduced cell growth in colorectal cancer cells.^77^ Table 2 provides a summary of the anti‐tumor effects of cannabinoids in different cancers.

**TABLE 2:** A summary of the anti‐tumor effects of cannabinoids in different cancers.

Cancer type	Cannabinoid	Experimental system	Effect	Reference
Lung carcinoma	THC	In vitro and in vivo (mouse)	Anti‐proliferative	51
	CBD	In vitro and in vivo (mouse)	Anti‐invasive and anti‐metastatic	7
Prostate carcinoma	AEA‐anandamide	In vitro	Anti‐proliferative	54
	CBD	In vitro and in vivo	Anti‐proliferative and apoptosis	74, 75
Breast carcinoma	CBD	In vitro and in vivo (mouse)	Anti‐proliferative, apoptosis, cell cycle arrest, and autophagy	6, 65, 66
Aggressive breast carcinoma	CBD	In vitro and in vivo	Inhibition of migration, invasion, and metastasis	6, 67, 68, 69
ErbB2‐driven metastatic breast carcinoma	THC	In vivo (mouse)	Anti‐proliferative, reduced lung metastases, inhibition of angiogenesis, apoptosis	55
Glioma	CBD	In vitro	Apoptosis, inhibition of cell migration and invasion	8, 58, 59, 60, 61, 62, 63, 64
		In vivo	Decreased tumor size, vascularization, growth and weight, induction of tumor regression	
Glioblastoma	CBG	In vitro	Anti‐invasive	76
Leukemias/lymphomas	CBD	In vivo and in vitro	Apoptosis	70, 71, 72, 73
Colorectal carcinoma	CBG	In vivo	Anti‐proliferative, apoptosis	77

The accumulated data show that non‐psychoactive cannabinoids such as CBD and CBG have illustrated a range of anti‐cancer effects in a multitude of different cancer cell lines. Therefore, CBD and CBG are attracting pharmacological interest due to their non‐psychotropic nature, ability to inhibit cancer cell proliferation, and induction of apoptosis.

## 9 THE ROLE OF CANNABINOIDS IN MEDIATION OF APOPTOSIS

Apoptosis is a form of programmed cell death that is essential for the development and survival of organisms.^78^, ^79^ Defects in the regulation of apoptotic cell death contribute to many diseases, including disorders in which cell accumulation occurs, such as cancer. The molecular machinery responsible for apoptosis has been revealed, uncovering a family of proteases, the caspases, which are accountable for the morphological and biochemical changes that characterize apoptosis.^80^, ^81^ Regulators of the caspases have also been identified including activators and inhibitors of these cell death proteases. Through the discovery of inputs from signal transduction pathways into the core of the cell death machinery, ways of linking environmental stimuli to cell death responses or maintenance of cell survival have been demonstrated.^81^ The characteristics of the apoptotic cell include chromatin condensation, nuclear fragmentation, plasma membrane blebbing, and cell shrinkage. Eventually, the cell breaks into small membrane‐surrounded fragments known as apoptotic bodies which are cleared by phagocytosis without provoking an inflammatory response. Understanding the molecular mechanisms of apoptosis provides insight into the causes of pathologies where abnormal cell death regulation occurs, such as in cancer, and is beginning to provide novel approaches to the treatment of human diseases.^81^

Cannabinoids have been shown to activate apoptosis through CB_1_ or CB_2_ receptors. New evidence has reported that CBD promotes cell death in various gastric cancer cell lines.^78^, ^79^, ^82^ CBD has been shown to induce apoptotic cell death by suppressing X‐linked inhibitor of apoptosis (XIAP), a well‐characterized anti‐apoptotic protein, in a dose‐ and time‐dependent manner. CBD inhibited XIAP by stimulating stress‐related genes of the endoplasmic reticulum (ER) in gastric cancer cells.^78^, ^79^ Zhang et al.^82^ showed that CBD treatment increased the protein levels of cleaved caspase‐3 and caspase‐9, subsequently inducing apoptosis cell death in gastric cancer cells. CBD increased Bax and decreased Bcl‐2 expression levels, causing a reduction of the Bcl‐2/Bax ratio. This in turn, determined an increase in mitochondrial membrane permeability and a decrease in mitochondrial transmembrane potential, thus allowing the release of cytochrome C into the cytosol and consequently, triggering apoptosis. Results from a recent study by Jeong et al.^78^, ^79^ suggest that CBD can cause Noxa‐induced cell death in colorectal cancer cells. They reported that Noxa, a pro‐apoptotic member belonging to the Bcl‐2 protein family, is important for CBD‐induced apoptosis. In this study, CBD induced apoptotic cell death via ROS/ER stress‐regulated Noxa activation. Treatment with CBD increased Noxa in a dose‐ and time‐dependent manner. Noxa stimulated ROS production, which further exacerbated apoptosis. Endocannabinoids such as AEA‐anandamide and 2‐AG have shown to produce a dose‐dependent cell growth inhibitory effect in prostate cancer cells via the activation of CB_1_ receptors. Using Annexin V assays, it was shown that endocannabinoids induced apoptosis causing an increase in the levels of activated caspase‐3 and a reduction in the levels of Bcl‐2. In addition, endocannabinoid treatment activated the ERK pathway and simultaneously produced a decrease in the activation levels of the AKT pathway.^83^ Apoptosis by cannabinoids is not exclusively carried out by CB_1_ and CB_2_ receptors. Endocannabinoids such as AEA‐anandamide have been shown to induce apoptosis through transient receptor potential vanilloid subtype 1 (TRPV1) activation in human neuroblastoma and lymphoma cells. This effect occurred through oxidative stress, increased calcium influx, and activation of caspases 3 and 9.^84^, ^85^ Furthermore, in cholangiocarcinoma cell lines, AEA‐anandamide exerted pro‐apoptotic activity, through the activation of GPR55 and induced apoptosis by the recruitment and activation of the death complex Fas/FasL.^86^

## 10 ECS IN THE OVARIES AND THEIR INTERACTIONS WITH FEMALE HORMONES

Studies have shown that CBD induced a reduction in cell proliferation in epithelial ovarian cancer cells, Kuramochi cell lines. Results from this study demonstrated that Kuramochi cell proliferation was 15% that of controls at CBD concentrations of 40 and 50 μM.^87^

CB_1_ receptors have been identified in normal, non‐cancerous ovaries.^88^ The presence of CB_2_ receptors has also been reported in the ovarian cortex, ovarian medulla, and ovarian follicles from human samples.^88^ Research has shown that expression of cannabinoid receptors changes as cancer progresses, specifically in ovarian cancer. Messalli et al. determined CB_1_ receptor expression in 66 human epithelial ovarian tumors and concluded that CB_1_ expression increased from benign and borderline to malignant tumors. They hypothesized that while low levels of cannabinoids may activate proliferative pathways in non‐cancerous cells, a higher cannabinoid concentration results in anti‐proliferative and apoptotic events in cancerous cells.^89^ Expression of the CB_1_ and CB_2_ receptors in cancerous cells, including the increase which was reported by Messalli et al. does not necessarily correlate with the expression pattern of the healthy tissue of origin.^90^ It is known that cannabinoid receptors and their endogenous ligands are generally upregulated in cancerous cells compared to non‐cancerous cells. Increased levels of cannabinoid receptor expression suggest that the administration of exogenous cannabinoids may impair tumor progression by inducing apoptosis.^21^, ^90^ It is clear that the expression of cannabinoid receptors varies but these contradictory observations highlight the gap in the knowledge of the mechanism behind the regulation of cannabinoid receptors in malignancy. By achieving clarity in the regulation of the CB_1_, CB_2_, and GPR55 receptors at different stages and grades of the disease, this will aid in a more accurate understanding of the mechanism of action of non‐psychoactive cannabinoids on these receptors in ovarian cancer cells.

It can be argued whether the change in expression of cannabinoid receptors and their endogenous ligands such as endocannabinoids is causing cancer or if this change occurs as a result of cancer progression. A study by Hofman et al. investigated the involvement of the cancer cell‐derived GPR55 receptor agonist, L‐α‐lysophosphatidylinositol (LPI), on angiogenesis in ovarian cancer cell lines. They found that the GPR55 receptor agonist, L‐α‐lysophosphatidylinositol, mediated angiogenesis as pharmacological inhibition of GPR55 reduced the pro‐angiogenic potential of L‐α‐lysophosphatidylinositol in these cell lines. Interestingly, LPI activated only GPR55 receptors and not CB_1_ and CB_2_ receptors.^91^, ^92^ Therefore, it can be concluded that inhibiting the pro‐angiogenic L‐α‐lysophosphatidylinositol/GPR55 pathway may be a promising target against angiogenesis in ovarian cancer.^93^ The expression of different components of the ECS is not uniform across all cancers; therefore, pharmacological manipulations of the ECS in further studies allow investigation into the link between the ECS and cancer progression.^89^, ^94^

Events in the ovarian cycle are controlled by hormones secreted by the hypothalamus, the anterior pituitary, and the ovaries, collectively known as the hypothalamic–pituitary‐gonadal axis.^95^ The ECS has been closely linked to the hypothalamic–pituitary‐gonadal axis. CB_1_ receptors have been identified in the hypothalamus and anterior pituitary and CB_1_ and CB_2_ receptors are present in the ovaries.^88^, ^96^, ^97^ In multiple studies, cannabinoids such as WIN‐55, AEA‐anandamide, and THC have been shown to reduce the release of gonadotropin‐releasing hormone (GnRH) through a variety of mechanisms. For example, by indirectly modifying GnRH release by reducing the activity of neurotransmitters that facilitate GnRH release such as glutamate, whilst stimulating the activity of those known to down‐regulate GnRH secretion such as GABA, as well as by directly inhibiting hypothalamic release of GnRH.^97^, ^98^, ^99^, ^100^, ^101^, ^102^, ^103^ This reduction in GnRH, in turn, causes decreased release of luteinizing hormone (LH) and follicle‐stimulating hormone (FSH) from the anterior pituitary, resulting in decreased release of estrogen from the ovaries.^104^ Therefore, it is evident that endocannabinoids down‐regulate hypothalamic–pituitary‐gonadal activity and consequently, cause a reduction in estrogen levels.

Not only has research highlighted the effects of cannabinoids on gonadal hormones such as estrogen, but studies have also demonstrated the effects of estrogen on the ECS. In a study by MacCarrone et al., it was shown that in mouse uterus, estrogen decreased the activity of FAAH, the major enzyme responsible for the degradation of the endocannabinoid AEA‐anandamide, and, therefore, led to increased concentrations of cannabinoid.^84^, ^85^ In another study, the administration of estradiol in ovariectomized female rats increased the levels of synthesized AEA‐anandamide in the medial basal hypothalamus indicating that estradiol could also increase endocannabinoid levels by directly interacting with endocannabinoid synthesis.^102^ Furthermore, another study showed that there was a positive correlation between the plasma peak levels of AEA‐anandamide and peak plasma 17β‐estradiol and gonadotrophin levels at ovulation.^105^ A possible underlying mechanism responsible for this could be that elevated levels of estrogens at ovulation inhibit FAAH activity, and as a consequence, increase plasma levels of endocannabinoids. Taken together, these studies show that, unlike the down‐regulation of hypothalamic–pituitary‐gonadal activity by endocannabinoids, it is evident that estrogen plays a role in modulating endocannabinoid signaling by up‐regulating cannabinoid content.^106^

Considering the well‐documented interactions between the ECS and gonadal hormones such as estrogens and the fact that these studies show that estrogen can modify cannabinoid levels and vice versa, it is not evident that the manipulation of the level of estrogen and, therefore, modification of the level of endocannabinoids may contribute to the progression of cancer. For example, in women who develop cancer while taking hormone‐replacement therapy or contraceptive pills, it could be argued that the manipulation of estrogen levels and therefore modification in the level of endocannabinoids could contribute to the progression of cancer. Although there is evidence that shows an interaction between estrogen and the ECS, more studies are required to investigate the link between estrogen manipulation and modification of endocannabinoid levels and the contribution of this to the development of gynecological cancers. This would ultimately enable us to establish the therapeutic potential of targeting the estrogen and ECS interaction as an approach for the treatment of gynecological cancers.

## 11 CANNABINOIDS IMPLICATION IN CELL DEATH THROUGH APOPTOSIS, CELL CYCLE ARREST, AND AUTOPHAGY IN OVARIAN CANCER

Research has highlighted key proteins and pathways involved in apoptosis that have been altered to evade cell death in ovarian cancer. Through comparing caspase‐3 and caspase‐8 levels in normal ovary, benign mass, and ovarian cancer, in vitro studies showed that caspase‐3 and caspase‐8 levels were lower in the benign mass and malignant group compared to the normal ovary group.^107^ Similarly, another study also showed that ovarian cancer tumors had low levels of caspase‐8 and were associated with shorter overall survival compared to tumors from patients that had high levels of caspase‐8.^108^ Therefore, since ovarian tumors have been shown to exhibit lower levels of these caspases, activating this pathway and thus increasing the levels of these caspases could provide a therapeutic strategy for inducing apoptosis in ovarian cancer cells. Survivin, a member of the Inhibitors of Apoptosis Proteins (IAP) family, blocks apoptosis by inhibiting caspase‐3 and caspase‐7. The over‐expression of survivin and, therefore, increased inhibition of caspase‐3 and caspase‐7 in ovarian cancer cell lines, IGROV‐1 and OAW42, highlighted its role in influencing cell sensitivity to taxanes.^109^ Histone deacetylase (HDAC) enzymes are a group of enzymes that are known to silence genes via catalyzing the removal of acetyl groups from histones as well as non‐histone proteins.^110^ In ovarian cancer, HDAC6 is often elevated leading to the inactivation of *p53* apoptotic function. This was reversed when Bitler et al. used a small molecule HDAC6 inhibitor, ACY1215.^111^ The PI3K/AKT pathway is a key intracellular signal transduction pathway and has an important role in the regulation of apoptosis and cell survival. The loss of *PTEN* together with other mutations causes this pathway to be over‐expressed, resulting in reduced apoptosis.^112^, ^113^ Enhanced expression of the PI3K/AKT pathway has been recognized as a hallmark of many cancers, including ovarian cancer.^114^

Key proteins involved in the regulation of cell cycle arrest have been identified to be exploited in ovarian cancer to promote cell cycle progression and inhibit cell cycle arrest. Cyclin E1 mainly coordinates with cyclin‐dependent kinase 2 (CDK2) to facilitate the progression of the G1/S cell cycle.^115^ In ovarian cancer cells, enforcing cyclin E1 expression stimulates cell proliferation,^116^ and over‐expression of cyclin E1 has been linked to the development of chemo‐resistance in ovarian cancer.^117^, ^118^ It has been shown that ovarian tumors with elevated cyclin E1 levels often exhibit higher CDK2 expression.^119^, ^120^ Studies show that the abundance of cyclin E1 correlates with tumor progression in patients with ovarian cancer.^121^, ^122^, ^123^, ^124^ Mutations in the tumor suppressor gene, *p53*, have been detected in all histological types of epithelial ovarian cancer, and serous carcinomas, have been detected at higher frequencies. Alterations in the *p53* network represent up to 96% of patients with high‐grade serous ovarian carcinoma.^125^ The loss of *p53* function is another exploited mechanism that ovarian cancer cells deploy to inhibit cell cycle arrest and apoptosis. Dysregulation of the cell cycle signaling pathway CDK4/6‐cyclin D/p16‐Rb is one of the most common abnormalities in human cancer.^126^ Studies have identified that in ovarian cancer, p16 expression is most commonly altered due to promoter methylation.^127^, ^128^, ^129^ Overexpression of cyclin D1 has been described in ovarian cancer tumors and has been associated with a more aggressive tumor phenotype and poor prognosis.^130^ Mutations of the *Rb* gene have been reported in ovarian cancers.^131^, ^132^ Together, these altered proteins and signaling pathways involved in the cell cycle regulation contribute to uncontrolled cell proliferation in ovarian cancer by avoiding cell cycle arrest.

Autophagy, from the Greek, meaning self‐eating refers to a cellular process committed to the lysosomal degradation of self‐constituents.^133^ Dysregulation of autophagy plays a significant role in the pathogenesis and resistance to radiotherapy and chemotherapy in ovarian cancer. A plethora of signaling pathways and proteins whose expression has been found altered in ovarian cancers have an impact on autophagy.^134^ For example, alterations in LC3, a component of the autophagy machinery. Compared to benign tissues and borderline ovarian tumors, highly malignant ovarian cancer cells were shown to express very low levels of LC3.^135^ Another example is an alteration in the signaling pathway, PI3K‐AKT‐mammalian target of rapamycin (mTOR). In ovarian cancer patients, a hyperactive state of mTOR has been associated with a poor prognosis.^136^ There is also sustained up‐regulation of the PI3K‐AKT–mTOR pathway in ovarian cancers that results in increased suppression of the autophagic process.^137^, ^138^ Moreover, deletion of the gene that encodes Beclin‐1, another component of the autophagy machinery, has been identified in ovarian cancers.^139^ The expression of Beclin‐1 has been shown to be dramatically higher in benign and borderline ovarian tumors than those in malignant ovarian cancers.^135^ Inhibition of autophagy contributed to ovarian cancer development and was required to suppress Beclin‐1 and up‐regulate Bcl‐2.^140^

**FIGURE 1:**
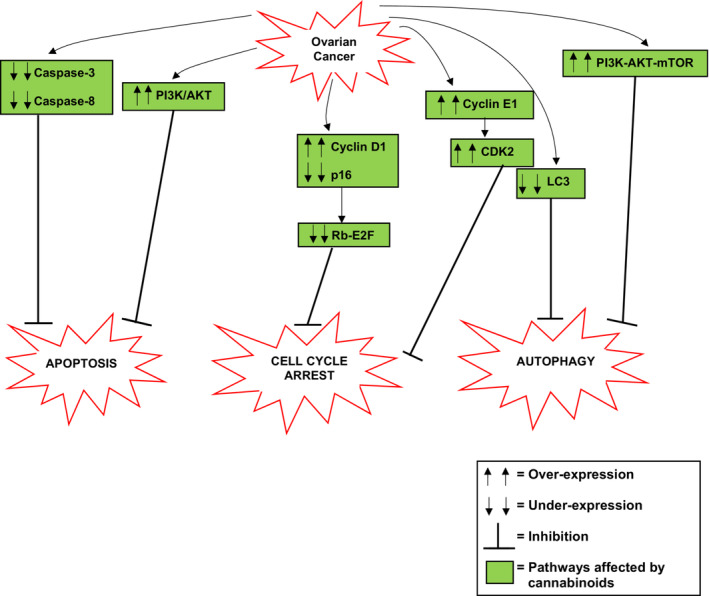
A summary of the altered signaling pathways and proteins leading to evasion of cell death via apoptosis, cell cycle arrest, and autophagy in ovarian cancer that are affected by cannabinoids.

From the accumulated literature it is evident that cannabinoids affect many of the pathways and proteins involved in the evasion of apoptosis, cell cycle arrest, and autophagy in ovarian cancer (Figure 1). For example, in ovarian cancer, there are reduced levels of caspase‐3 and caspase‐8 contributing to inhibition of apoptosis. Cannabinoids can activate these caspases and thus activate apoptosis.^4^ The increased expression of the PI3K/AKT pathway contributing to inhibition of apoptosis in ovarian cancer cells is a pathway that is affected by cannabinoids. Cannabinoids can inhibit this pathway leading to activation of apoptosis. Some of the key proteins and pathways involved in cell cycle arrest that have been exploited in ovarian cancer to enable cell survival have also been shown to be affected by cannabinoids. For example, the increased activation of cyclin D leads to loss of Rb‐E2F, and thus inhibition of cell cycle arrest in ovarian cancer is a pathway that is affected by cannabinoids. Through activation of p27, cannabinoids can inhibit cyclin D that leads to activation of Rb‐E2F and therefore activation of cell cycle arrest. Similarly, the increased activation of cyclin E and, consequently, increased activation of CDK2 in ovarian cancer are proteins that are affected by cannabinoids. Through multiple mechanisms, cannabinoids can inhibit the activity of cyclin E and CDK2 and therefore allow activation of cell cycle arrest. It has also been shown that cannabinoids can affect some of the key proteins and pathways involved in autophagy that have been exploited in ovarian cancer to enable cell survival. One of these proteins being LC3. The reduced levels of LC3 in ovarian cancer inhibit autophagy, however, cannabinoids such as CBD and AEA‐anandamide have been shown to activate this protein, resulting in the activation of autophagy. Another pathway that cannabinoids can affect is the PI3K‐AKT–mTOR pathway. In ovarian cancer, this pathway is upregulated as a mechanism of inhibiting autophagy, however, it has been shown that cannabinoids inhibit this pathway leading to activation of autophagy. Therefore, the accumulated data indicate that cannabinoids have a role in the mediation of cell death through apoptosis, cell cycle arrest, and autophagy in ovarian cancer and could provide a therapeutic strategy that targets the ability to evade cell death in ovarian cancer (Figure 1).

## 12 CONCLUSION AND FUTURE DIRECTION

The current clinical status of gynecological cancers inevitably motivates researchers to identify and explore new and improved therapies for patients. Pre‐clinical studies suggest that cannabinoids may exert favorable outcomes in the treatment of cancers. Although the presence of psychoactive effects limits the progression of cannabinoids into clinical practice, the use of non‐psychoactive cannabinoids that are devoid of these adverse effects such as CBD provides a solution.

The anti‐proliferative and pro‐apoptotic effects of cannabinoids are well established. However, the relationship between the effects of cannabinoids and the expression of cannabinoid receptors in gynecological malignancy needs further investigation. The introduction of cannabinoid‐based medications such as sativex and epidiolex (CBD) is encouraging. Currently, sativex and epidiolex are the only cannabinoid‐based medications that are licensed for use in the UK (British National Formulary, 2022).^141^ Sativex is available in the form of an oromucosal spray and contains both THC and CBD in an approximate 1:1 ratio. Sativex is indicated for adjunctive therapy in the treatment of moderate to severe spasticity in multiple sclerosis (Electronic Medicines Compendium, 2022).^142^ Epidiolex is in the form of an oral solution and contains CBD. Epidiolex is indicated for adjunctive therapy in the treatment of seizures associated with Lennox–Gastaut syndrome or Dravet syndrome to be used in conjunction with clobazam. Epidiolex is also indicated for adjunctive therapy in the treatment of seizures associated with the tuberous sclerosis complex (Electronic Medicines Compendium, 2022).^143^ Both drugs were well tolerated with minimum and manageable side effects.^144^, ^145^, ^146^, ^147^, ^148^, ^149^, ^150^, ^151^ This has encouraged a UK randomized clinical trial to test the use of sativex in patients with glioblastoma. The trial is aimed at measuring whether adding sativex to chemotherapy extends overall patient survival, delays the progression of the disease, or improves quality of life.

With the encouraging pre‐clinical results on anti‐tumor effects of cannabinoids in ovarian cancer, further research is needed to complement the existing knowledge and enable the translation of cannabinoids for cancer treatment into the clinic. This should be followed by the initiation of clinical trials to re‐purpose sativex and epidiolex in ovarian cancer patients.

In addition, future work should aim to understand the change in the level of estrogen on the level of the cannabinoid system and vice versa in gynecological cancers. Nevertheless, the accumulated data support further studies on the use of cannabinoids as a potential candidate in the treatment strategy for ovarian cancer. This review serves as a platform upon which existing knowledge and research can be built upon to ultimately establish if non‐psychoactive cannabinoids have the potential of becoming a part of an effective ovarian cancer strategy.

## AUTHOR CONTRIBUTIONS

Both authors contributed to all aspects of writing.

## CONFLICT OF INTEREST STATEMENT

The authors declare that they have no conflict of interest.

## ETHICS STATEMENT

Not applicable.

## Data Availability

Not applicable.
